# Viscosity estimation model of fluorine-containing mold flux for continuous casting

**DOI:** 10.1371/journal.pone.0247828

**Published:** 2021-03-05

**Authors:** Zhongyu Zhao, Junxue Zhao, Boqiao Qu, Yaru Cui

**Affiliations:** 1 School of Metallurgical Engineering, Xi’an University of Architecture and Technology, Xi’an, Shaanxi, China; 2 CISDI Research & Development Co. Ltd., Chongqing, China; University of Vigo, SPAIN

## Abstract

A viscosity estimation model for fluorine-containing mold flux for continuous casting was investigated based on the Arrhenius formula and the rotating cylinder method combined with nonlinear regression analysis. This model is highly applicable and not limited by the slag of a certain composition. For most slag compositions, the viscosities estimated with this model deviated from the measured values by no more than 10%, which was in better agreement with the measured values than the viscosities estimated by the Riboud, Iida and Mills models. According to the model calculation and experimental detection, a viscosity isogram of CaF_2_-Na_2_O-Al_2_O_3_-CaO-SiO_2_-MgO slag was produced, and the mass fraction of CaF_2_ in the low-viscosity zone was nearly 14%. An X-ray fluorescence spectrometric analysis of slag after the viscosity test showed that CaF_2_ and Na_2_O were significantly reduced, and the measured viscosity was greater than the theoretical viscosity due to the volatilization.

## Introduction

The fluidity of slag has an important influence on the refining reaction, smelting temperature control, and heat and mass transfer of slag and metal during the steelmaking process [1–5]. Viscosity is the main factor affecting the fluidity of slag, and many scholars have analyzed the influence factors and control mechanisms of various kinds of slag viscosity with the objective of securing a universally applicable model to predict the viscosities of different kinds of slag changes under different temperatures [6–15]. The Riboud [16] proposed a viscosity model based on the Weymann Frenkel formula. Iida [17] put forward the viscosity model combined with crystallization temperature, and Mills [18] built a viscosity model based on optical basicity that widely used to estimate the viscosity of mold flux during continuous casting. However, each method has a certain limitation and scope of application, and there are significant differences between the viscosity estimations and the test results, especially for slag containing fluorine.

This article put forward a new viscosity model based on the Arrhenius equation and nonlinear regression analysis considering comprehensively the volatilization of fluorine-containing mold flux for continuous casting. An isoviscosity diagram is drawn according to this model and can provide theoretical support and practical basis for slag composition design and performance control.

## Materials and methods

The composition of fluorine-containing mold flux was designed in Table 1, according to the requirements and application of the continuous casting technique.

**Table 1:** Designed composition of fluorine-containing mold flux.

Component	CaF_2_	R(CaO/SiO_2_)	Na_2_O	Al_2_O_3_	MgO
Mass fraction/%	4~20	0.6~1.2	3~12	2~12	0~12

Twenty-seven viscosity tests (C1 ~ C27) were designed by quadratic regression orthogonal analysis [19] to obtain a viscosity estimation model based on the Arrhenius formula. To evaluate the effectiveness and applicability of this model, the viscosity data of fluorine-containing mold fluxes at the American National Physical Laboratory and the Department of Theoretical Metallurgy (M1 ~ M11) [20] were obtained and analyzed. At the same time, combined with the viscosity data of CaF_2_-CaO-SiO_2_ slag (S1–S7) [21], the uncertain relation of viscosity detection and slag volatilization was investigated. The compositions of the slags are given in Table 2.

**Table 2:** Components of mold fluxes for continuous casting, wt %.

Slag	CaO	SiO_2_	Al_2_O_3_	CaF_2_	Na_2_O	MgO	(R)	Slag	CaO	SiO_2_	Al_2_O_3_	CaF_2_	Na_2_O	MgO	(R)
C1	24.6	22.3	11.2	19	11.7	11.2	1.1	C24	33.7	37.4	7.2	12.4	3.1	6.2	0.9
C2	34.0	30.9	10.5	17.8	4.7	2.1	1.1	C25	27.6	30.7	7.6	13	8.1	13	0.9
C3	35.1	32.0	11.2	7.8	11.7	2.2	1.1	C26	33.8	37.5	7.6	13	8.1	0	0.9
C4	35.1	31.9	10.5	7.3	4.7	10.5	1.1	C27	30.7	34.1	7.6	13	8.1	6.5	0.9
C5	32.8	29.8	4.5	19	11.7	2.2	1.1	M1	23.4	38.3	6.2	12.9	18.8	0.5	0.6
C6	32.9	29.9	4.2	17.8	4.7	10.5	1.1	M2	17.5	40.0	5.2	14.5	21.5	1.3	0.4
C7	33.9	30.9	4.5	7.8	11.7	11.2	1.1	M3	20.1	34.4	4.8	14.3	26.4	0.0	0.6
C8	42.8	38.9	4.2	7.3	4.7	2.1	1.1	M4	19.4	36.6	18.0	17.2	8.8	0.0	0.5
C9	23.0	32.9	11.2	19	11.7	2.2	0.7	M5	32.6	31.2	5.3	8.0	22.9	0.0	1.0
C10	23.3	33.2	10.5	17.8	4.7	10.5	0.7	M6	22.5	42.2	10.4	10.7	13.0	1.3	0.5
C11	23.9	34.2	11.2	7.8	11.7	11.2	0.7	M7	21.5	33.2	3.7	15.4	25.7	0.5	0.6
C12	31.0	44.4	10.5	7.3	4.7	2.1	0.7	M8	22.7	38.6	6.3	13.5	18.9	0.0	0.6
C13	22.1	31.5	4.5	19	11.7	11.2	0.7	M9	22.3	35.0	8.9	16.5	17.3	0.0	0.6
C14	29.3	41.9	4.2	17.8	4.7	2.1	0.7	M10	20.4	34.0	4.3	17.9	23.4	0.0	0.6
C15	30.4	43.4	4.5	7.8	11.7	2.2	0.7	M11	13.8	32.0	3.5	20.2	27.1	3.5	0.4
C16	30.2	43.1	4.2	7.3	4.7	10.5	0.7	S1	33.5	53.0	-	13.5	-	-	0.6
C17	35.3	29.5	7.6	13	8.1	6.5	1.2	S2	32.9	47.8	-	19.3	-	-	0.7
C18	24.3	40.5	7.6	13	8.1	6.5	0.6	S3	33.1	46.9	-	20.0	-	-	0.7
C19	28.1	31.3	13	13	8.1	6.5	0.9	S4	30.8	37.8	-	31.4	-	-	0.8
C20	33.3	36.9	2.2	13	8.1	6.5	0.9	S5	27.0	30.4	-	42.6	-	-	0.9
C21	26.6	29.6	7.6	21.6	8.1	6.5	0.9	S6	33.0	39.6	-	27.4	-	-	0.8
C22	34.8	38.7	7.6	4.3	8.1	6.5	0.9	S7	33.2	38.6	-	28.2	-	-	0.9
C23	27.5	30.5	8	13.6	13.6	6.8	0.9								

(Note: the wt% Na_2_O of samples M1 to M11 corresponds to the sum of wt% Na_2_O and wt% K_2_O)

The slags (C1 ~ C27) were prepared with chemical reagents according to Table 2, and the samples were ground by an agate ball mill at a speed of 200 r/min for 0.5 h, dried at 373 K for 5 h in GF101-2A Electric Blast Drying Oven, and then sealed and stored in the dark. An RTW-10 Melt Physical Property Comprehensive Analyzer was used for the viscosity experiment, and the heating rate was 10 K/min. It was cooled at 5 K/min when the temperature was reached to 1773 K, and the viscosity was recorded. High purity argon was used to protect the detection process, and the flow rate was set at 50ml / min.

## Results and discussion

### Viscosity analysis

**Fig 1:**
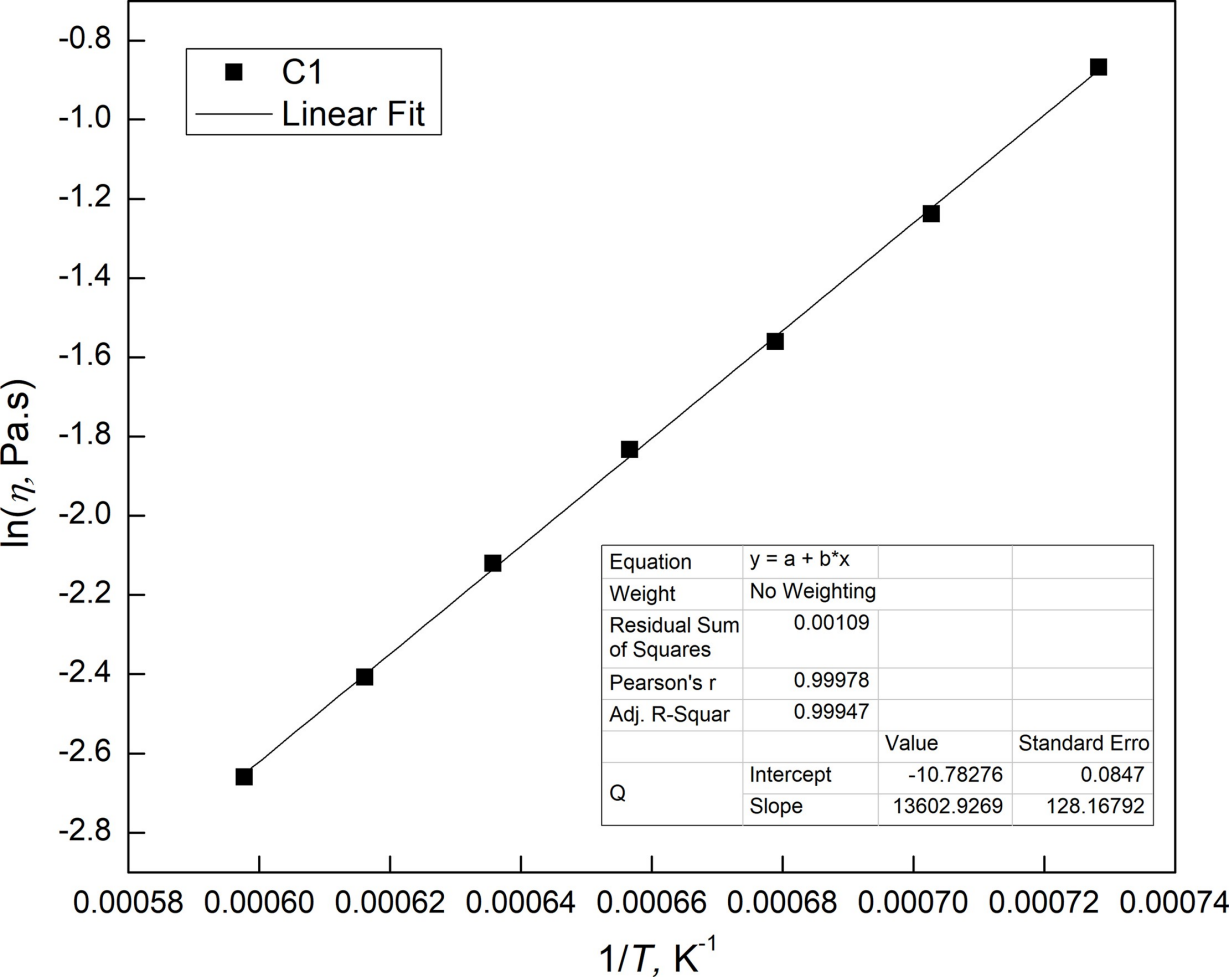
Linear analysis of ln*η* to 1/*T*.

Viscosity tests of the C1 ~ C27 slag samples were performed to obtain the linear relationship between the logarithm of viscosity (ln *η*) and the reciprocal of temperature (1/*T*) based on the Arrhenius equation (formulas 1 ~ 2). Taking the C1 slag viscosity test as an example, the results of the analysis are shown in Fig 1. The intercept (ln *A*) and slope (*B* = *E/*R) of each linear relationship were obtained in Table 3.

**Table 3:** Viscosity controlling parameters.

Slag	ln*A*	*B*	Slag	ln*A*	*B*	Slag	ln*A*	*B*
C1	-10.7828	13602.93	C10	-11.1663	15523.76	C19	-11.6419	16899.2
C2	-11.1521	15331.25	C11	-11.4069	16399.51	C20	-10.5736	14696.67
C3	-11.4827	16323.8	C12	-11.9045	19019.28	C21	-9.26063	11473.14
C4	-11.2338	16106.31	C13	-10.7114	13670.59	C22	-11.3388	18048.45
C5	-10.9666	13904.19	C14	-11.1896	15932.56	C23	-10.4814	13697.65
C6	-11.1806	14859.89	C15	-11.4391	17000.89	C24	-17.2362	25991.79
C7	-11.3522	15328.48	C16	-11.1312	16491.46	C25	-10.5812	14320.87
C8	-11.3635	16421.96	C17	-14.961	21103.44	C26	-13.0682	18316.6
C9	-10.9992	14722.03	C18	-12.8972	19711.71	C27	-12.1166	16750.76

$$\eta =A\exp(\frac{E}{RT})$$ (1)

$$\ln\eta =\ln A+\frac{E}{R}\times \frac{1}{T}$$ (2)

The viscosity parameters ln *A* and *B* were obtained by nonlinear regression analysis according to changes in the basicity (R) and composition, and the parameter models were as follows.

$$\begin{array}{l} lnA=-6.69+0.07x_{1}-0.30x_{2}-0.93x_{3}+0.26x_{4}-0.08x_{5}+0.11x_{1}x_{2}+0.22x_{1}x_{3}-0.45x_{1}x_{4}-0.12x_{1}x_{5}\\ +0.05x_{2}x_{4}-0.01x_{2}x_{5}-0.02x_{3}x_{4}+0.01x_{3}x_{5}+0.01x_{4}x_{5}-0.05x_{1}{}^{2}-0.01x_{2}{}^{2}+0.04x_{3}{}^{2}+0.01x_{5}{}^{2} \end{array}$$ (3)

$$\begin{array}{l} B=17381.7-9875.3x_{1}+597.7x_{2}+1160.7x_{3}-456.8x_{4}+32.0x_{5}-229.1x_{1}x_{2}-234.3x_{1}x_{3}+758.5x_{1}x_{4}+367.4x_{1}x_{5}\\ +2.59x_{2}x_{3}-82.6x_{2}x_{4}+15.7x_{2}x_{5}+30.93x_{3}x_{4}-10.7x_{3}x_{5}-28.6x_{4}x_{5}+2772.7x_{1}{}^{2}+15.34x_{2}{}^{2}-52.1x_{3}{}^{2}-5.4x_{4}{}^{2}-22.1x_{5}{}^{2} \end{array}$$ (4)

**Fig 2:**
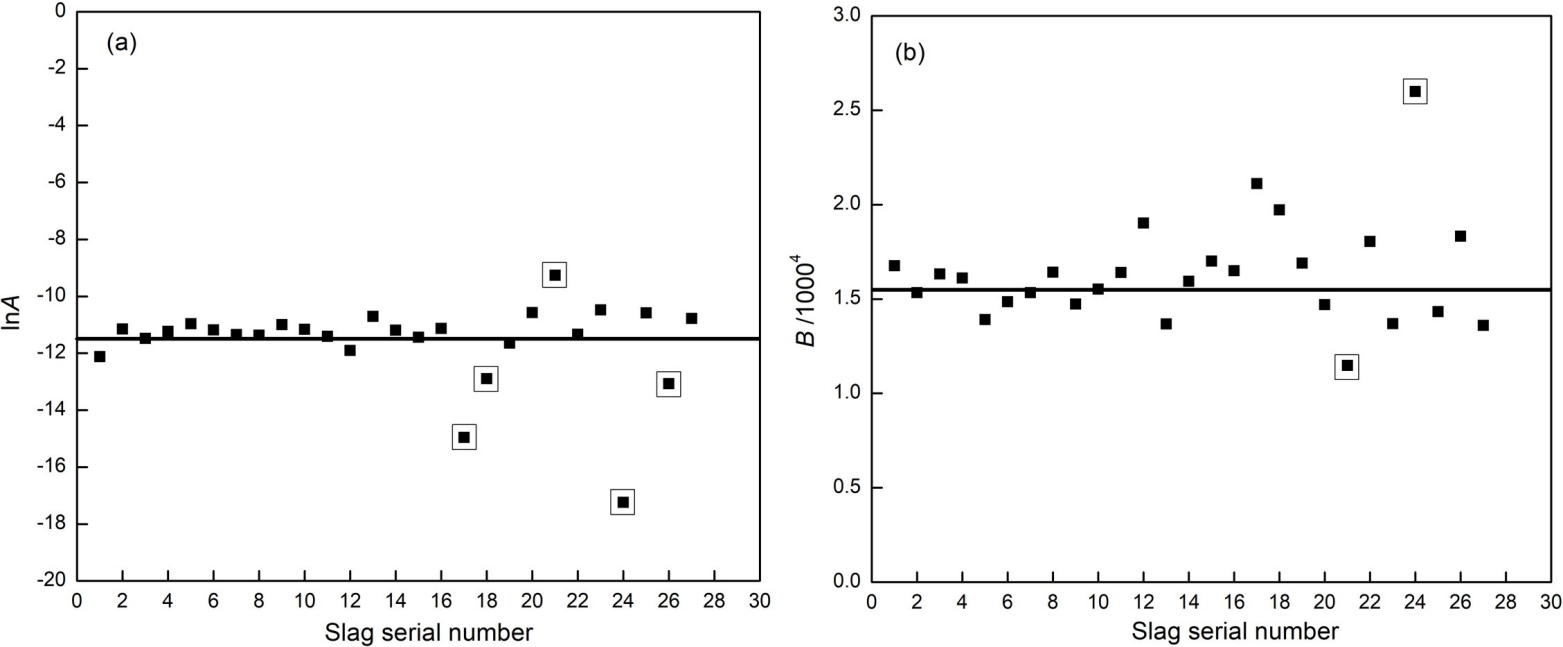
Viscosity parameter fitting value. (a) *lnA*; (b) *B*.

*x*_*1*_, *x*_*2*_, *x*_*3*_, *x*_*4*_, and *x*_*5*_ represent the basicity R and the mass fractions of Al_2_O_3_, CaF_2_, Na_2_O, and MgO, respectively. The above regression equations were analyzed with SPSS statistical software, for P = 0.0001 < 0.05, and correlation coefficient R = 0.9941, which showed a good fit. Fig 2 was drawn to clarify the influence of each component on the parameters *lnA* and *B*.

It can be seen from Fig 2A that *lnA* is between -10 and -12, and only five samples have relatively larger deviation, namely C17 [alkalinity]max, C18 [alkalinity]_min_, C21 [CaF_2_(wt%)]_max_, C24 [Na_2_O(wt%)]_min_, and C26 [MgO(wt%)]_min_. In particular, C21 corresponds to the largest *lnA*, C24 corresponds to the minimum *lnA*. The fitting points fluctuation in Fig 2B is opposite to that in Fig 2A. Therefore, CaF_2_ and Na_2_O are two significant factors effecting the viscosity of mold flux.

### Comparison of viscosity estimation models

**Fig 3:**
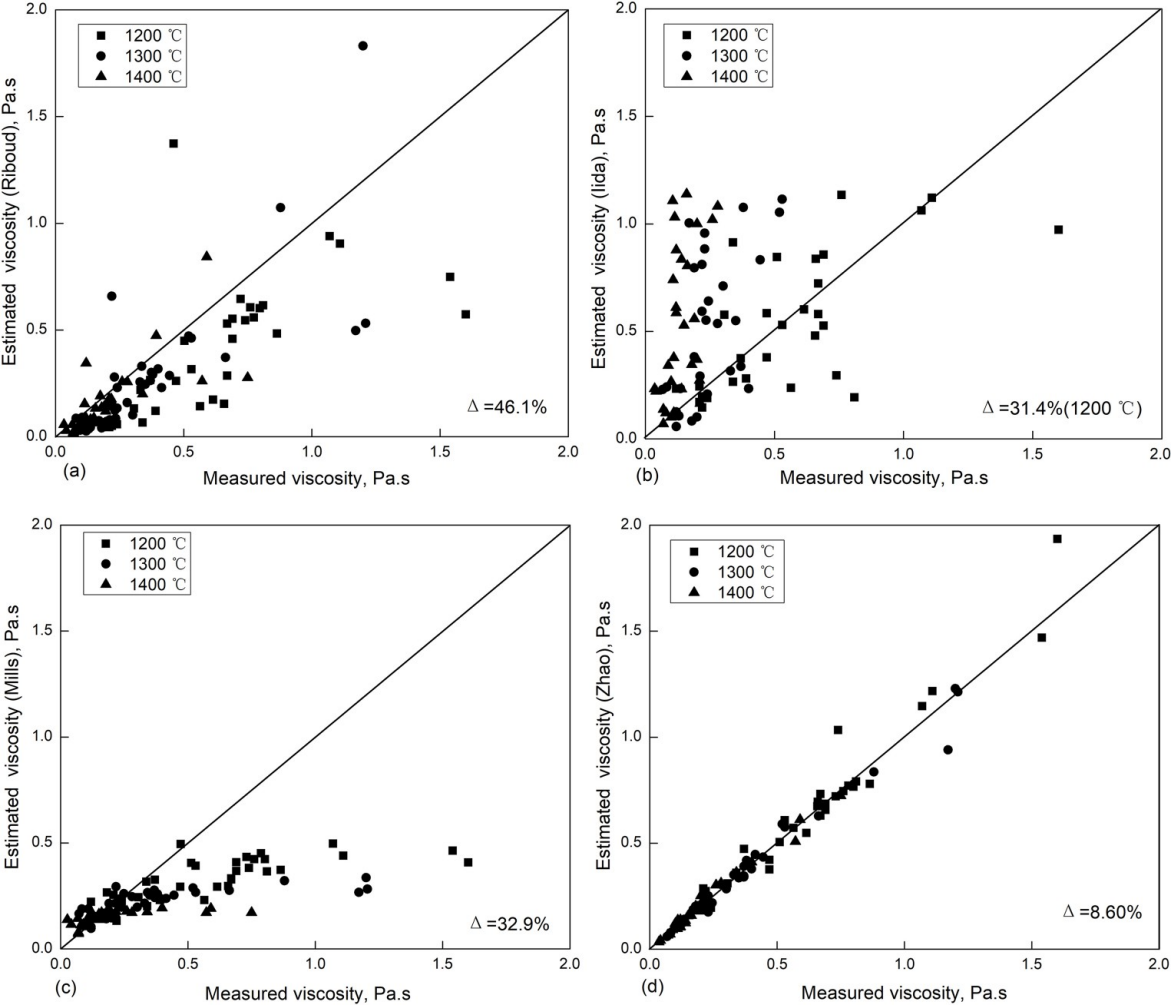
Estimated and measured viscosity of fluorine-containing mold flux. (a) Riboud model; (b) Iida model; (c) Mills model; (d) studied model. (the Δ in the figures above represents the deviation rate between the estimated viscosity and the measured viscosity, and the calculation formula (5) is shown below).

Combined with the viscosity tests (C1 ~ C27) and the viscosity data of fluorine-containing mold fluxes (M1 ~ M11), this viscosity estimation model can be evaluated and compared with the traditional viscosity models. The results are shown in Fig 3 below.

$$\Delta =\frac{1}{N}\sum_{n=1}^{N}\frac{{\left(\eta_{n}\right)}_{est}-{\left(\eta_{n}\right)}_{mea}}{{\left(\eta_{n}\right)}_{mea}}$$ (5)

In comparing the above models to estimate the viscosity of different types of fluorine-containing continuous casting mold fluxes, the deviation rate of the Riboud model was relatively larger. The estimated viscosity of the Iida model was higher than the measured viscosity, and the estimated value of the Mills model was lower than the measured value. In contrast, the deviation rate between the estimated and measured viscosity was less than 10%, and this model could better describe the viscosity change of different fluorine-containing continuous casting mold fluxes.

**Fig 4:**
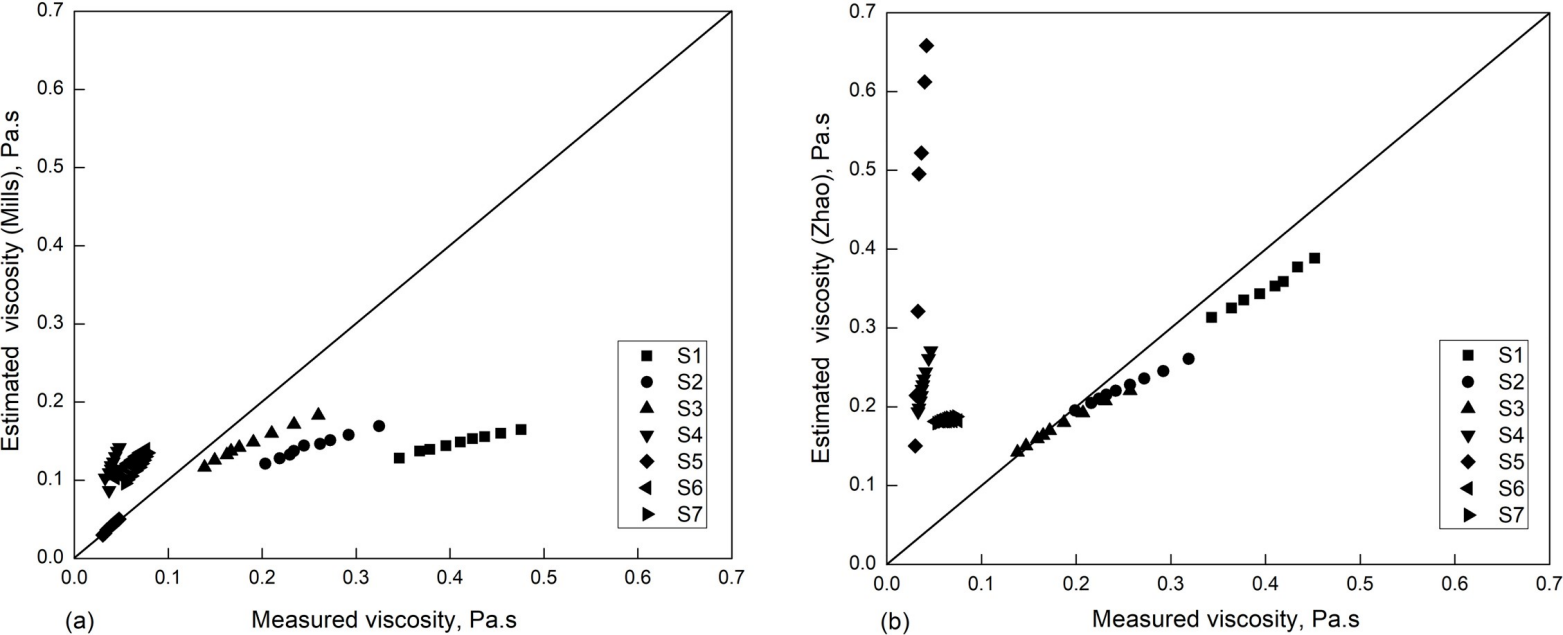
Estimated and measured viscosity of CaF_2_- CaO- SiO_2_ slag. (a) Mills model; (b) studied model.

At the same time, the viscosity data of CaF_2_-CaO-SiO_2_ slag (S1~S7) were compared and analyzed by the Mills model and Zhao model, respectively, as shown in Fig 4.

It can be seen that the Mills model had a better fitting on the viscosity of high fluorine content slag (S5), and the viscosity fitting by the Zhao model was better for medium and low fluorine content slag (S1 ~ S3). This was mainly affected by the volatility of the fluorine-containing slag; the higher the fluorine content is, the stronger the slag volatilization and the greater the change in composition, eventually leading to a deviation between the estimated and measured viscosity.

### Application of this viscosity estimation model

**Fig 5:**
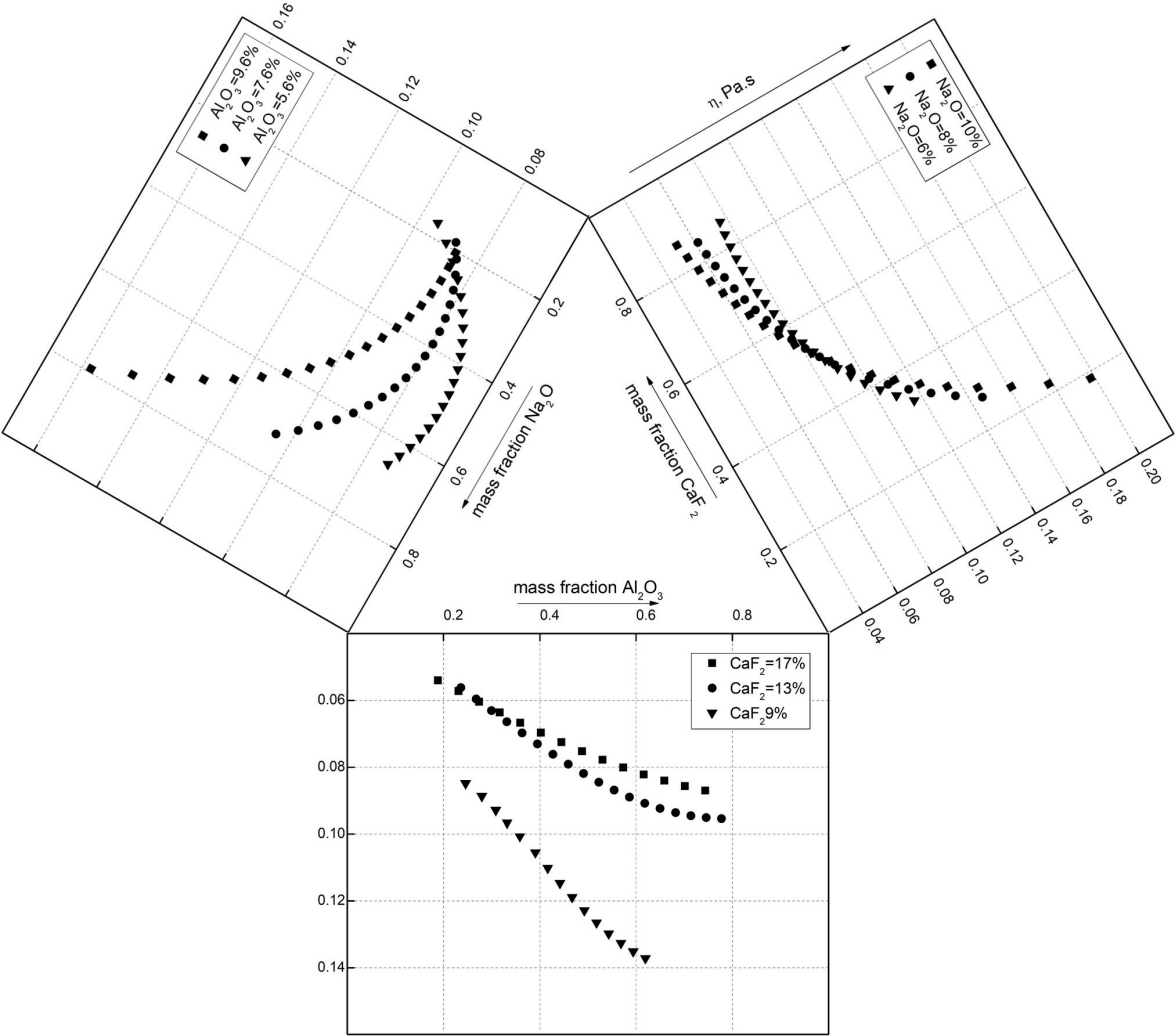
Effect of CaF_2_, Na_2_O and Al_2_O_3_ on the viscosity of mold flux at 1773 K.

The above viscosity estimation model can determine the influence of different component changes on the viscosity of the mold flux based on C27 slag with a fixed basicity R = 0.9 and MgO (wt%) = 6.5, and the CaF_2_, Na_2_O and Al_2_O_3_ components impact the slag viscosity in Fig 5.

It can be seen from the first part of Fig 5C that the increase in CaF_2_ can significantly reduce the viscosity of the mold flux. However, the influence of Al_2_O_3_ and Na_2_O on viscosity was restricted by the CaF_2_ content in the slag system. In Fig 5A, the CaF_2_ content is 17% at the intersection point. On the left side of the intersection, i.e. CaF_2_(wt%)<17%, the increase in Al_2_O_3_ significantly increased the viscosity of the slag. If the CaF_2_ content was more than 17%, the viscosity decreased with the addition of Al_2_O_3_. In Fig 5B, the CaF_2_ content is 11.5% at the intersection point. The viscosity of the slag system decreased significantly with increasing Na_2_O mass when the CaF_2_ content was more than 11.5%, and if the CaF_2_ content was less than 11.5%, the effect of Na_2_O on viscosity was insignificant.

**Fig 6:**
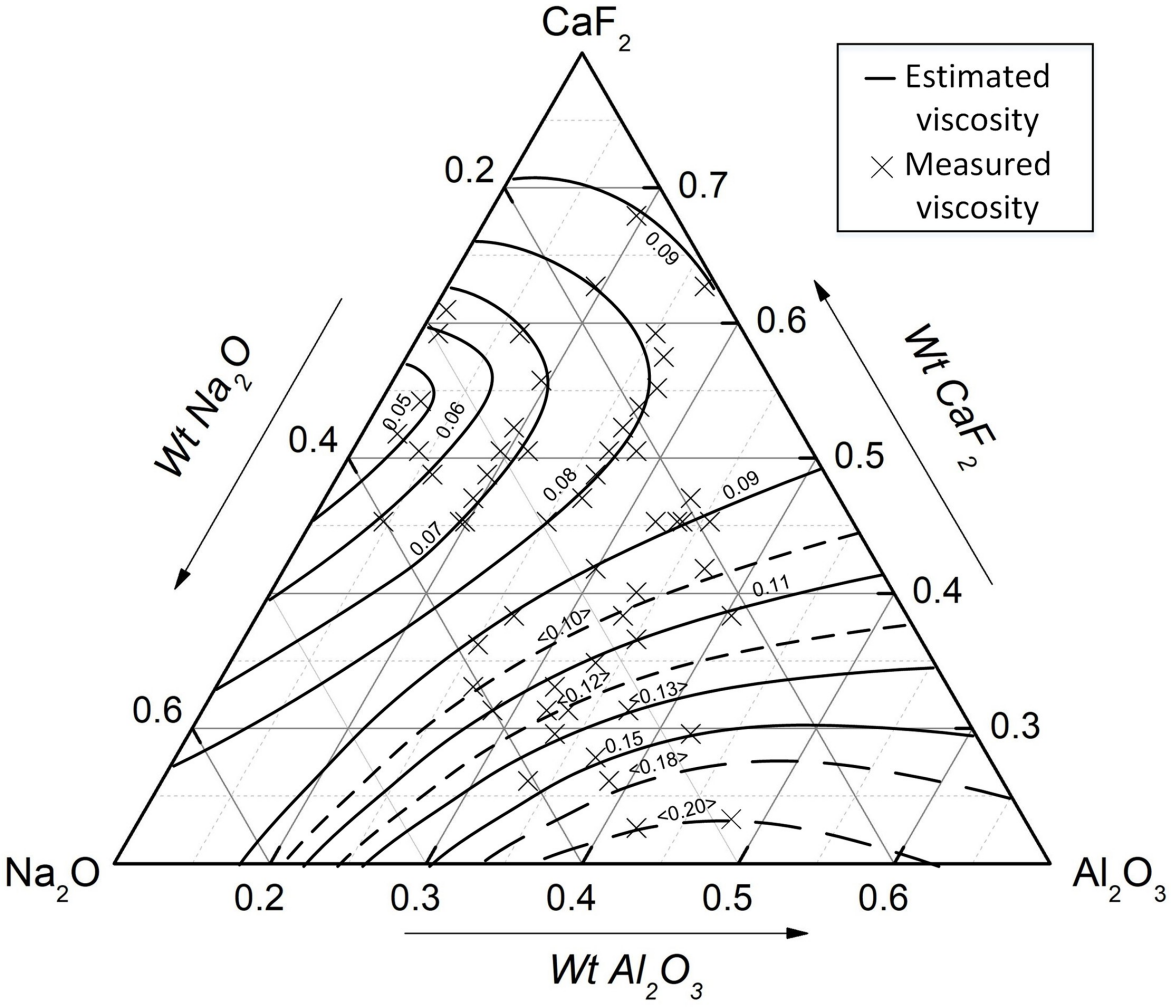
CaF_2_-Na_2_O-Al_2_O_3_-CaO-SiO_2_-MgO isoviscosity diagram (R = 0.9, MgO(wt%) = 6.5, 1773 K).

According to this viscosity estimation model and the experimental data, an isoviscosity diagram of CaF_2_-Na_2_O-Al_2_O_3_-CaO-SiO_2_-MgO at 1773 K can be drawn in Fig 6, similarly, with a fixed basicity R = 0.9 and MgO (wt%) = 6.5.

It can be seen from the isoviscosity curves in Fig 6 that the mass fraction of CaF_2_ in the low viscosity zone was nearly 14%, and a level of CaF_2_ that is too high or too low will increase the viscosity of the slag system. At the same time, the viscosity will increase gradually near the Al_2_O_3_ angle. The viscosity of the mold flux can be reduced by rationally adjusting the components according to the above viscosity isogram.

However, the volatilization leads to the change of slag composition, resulting in the measured viscosity cannot correspond to the initial composition of slag, as shown in Fig 4. It is necessary to detect the composition of samples after viscosity tests.

### Viscosity model modification based on volatilization

The composition of samples after viscosity tests was carried out by XRF analysis due to the volatilization of fluoride in the viscosity measurements, and the results are shown in Table 4.

**Table 4:** Components of mold fluxes after viscosity tests, wt %.

Slag	CaO	SiO_2_	Al_2_O_3_	CaF_2_	Na_2_O	MgO	(R)	Slag	CaO	SiO_2_	Al_2_O_3_	CaF_2_	Na_2_O	MgO	(R)
C1	29.9	23.6	11.9	14.7	8.1	11.9	1.3	C15	38.9	47.2	4.9	0.4	6.3	2.4	0.8
C2	38.6	32.3	11.0	14.3	1.5	2.2	1.2	C16	36.5	45.9	4.5	1.7	0.2	11.2	0.8
C3	41.1	33.8	11.8	2.9	8.1	2.3	1.2	C17	46.7	32.7	8.4	4.1	0.8	7.2	1.4
C4	41.8	34.0	11.2	1.7	0.1	11.2	1.2	C18	32.2	44.0	8.3	6.0	2.4	7.1	0.7
C5	40.4	32.1	4.8	13.4	7.0	2.4	1.3	C19	36.2	33.9	14.1	6.2	2.5	7.1	1.1
C6	38.3	31.5	4.4	13.7	1.0	11.1	1.2	C20	41.3	39.9	2.4	6.6	2.8	7.0	1.0
C7	39.2	32.5	4.7	3.4	8.5	11.8	1.2	C21	34.3	32.0	8.2	15.7	2.7	7.0	1.1
C8	50.0	41.5	4.5	1.7	0.1	2.2	1.2	C22	39.6	40.5	8.0	0.1	5.0	6.8	1.0
C9	29.0	35.1	11.9	14.1	7.6	2.3	0.8	C23	42.1	35.3	9.2	1.3	4.2	7.9	1.2
C10	28.4	35.2	11.1	13.5	0.7	11.1	0.8	C24	37.9	39.0	7.5	9.0	0.1	6.5	1.0
C11	28.5	35.9	11.8	3.5	8.6	11.8	0.8	C25	32.4	32.3	8.0	8.9	4.7	13.7	1.0
C12	36.3	46.7	11.1	2.7	1.0	2.2	0.8	C26	39.4	39.6	8.0	8.5	4.4	0.0	1.0
C13	26.0	32.9	4.7	15.8	9.0	11.7	0.8	C27	37.0	36.3	8.1	7.8	3.8	6.9	1.0
C14	35.5	44.6	4.5	12.9	0.2	2.2	0.8								

The viscosity model (1) ~ (2) can be modified according to the data in the table above, as shown below.

$$\begin{array}{l} lnA=-7.05-1.33x_{1}-1.08x_{2}-0.47x_{3}+0.43x_{4}-0.25x_{5}-0.35x_{1}x_{2}-0.32x_{1}x_{3}+0.56x_{1}x_{4}-0.2x_{1}x_{5}\\ -0.01x_{2}x_{3}+0.02x_{2}x_{4}-0.01x_{3}x_{5}+0.02x_{4}x_{5}+2.69x_{1}{}^{2}+0.09x_{2}{}^{2}+0.06x_{3}{}^{2}-0.16x_{4}{}^{2}+0.04x_{5}{}^{2} \end{array}$$ (6)

$$\begin{array}{l} B=11257.7+3200.1x_{1}+1942.2x_{2}+429.5x_{3}-1038.1x_{4}+405.8x_{5}+477.4x_{1}x_{2}+696.9x_{1}x_{3}-786.5x_{1}x_{4}+351.1x_{1}x_{5}\\ +16.9x_{2}x_{3}-42.1x_{2}x_{4}-4.4x_{2}x_{5}+0.9x_{3}x_{4}+22.7x_{3}x_{5}-20.3x_{4}x_{5}-6651.4x_{1}{}^{2}-141.4x_{2}{}^{2}-99.0x_{3}{}^{2}+262.5x_{4}{}^{2}-76.9x_{5}{}^{2} \end{array}$$ (7)

**Fig 7:**
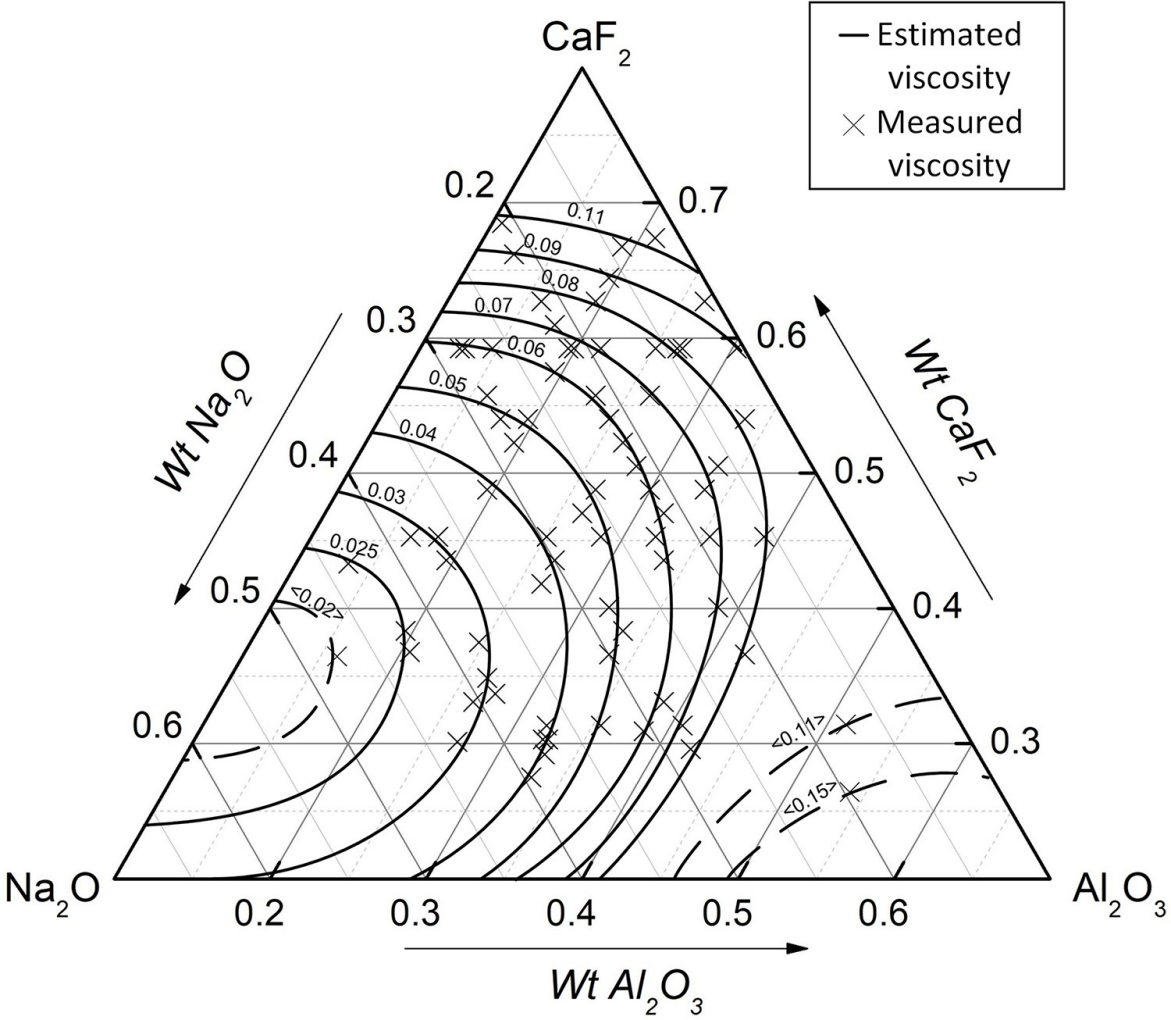
Modified isoviscosity diagram (R = 0.9, MgO(wt%) = 6.5, 1773 K).

Similarly, the isoviscosity diagram of mold flux at 1773 K can be drawn in Fig 7 based on the above model with a fixed basicity R = 0.9 and MgO (wt%) = 6.5.

Fig 7 shows the viscosity corresponding to the actual composition of the mold flux, and Fig 6 shows the viscosity corresponding to the initial composition. Comparing the isoviscosity curves in Figs 6 and 7, it can be seen that Na_2_O decreased significantly during the viscosity test. Moreover, Tables 2 and 4 show that CaF_2_ also decreased. Therefore, it can be seen that the measured viscosity in Fig 6 was relatively larger than the theoretical viscosity in Fig 7 due to volatilization.

## Conclusions and prospects

1. A viscosity estimation model of fluorine-containing mold flux for continuous casting was investigated through viscosity detection and nonlinear regression analysis based on the Arrhenius equation. The viscosities estimated with this model were within 10% of the measured values, which achieves better agreement with the measured values than the viscosities estimated by the Riboud, Iida and Mills models.
2. CaF_2_ can significantly reduce the viscosity of mold flux, and the influence of Al_2_O_3_ and Na_2_O on viscosity was restricted by the CaF_2_ content in the slag system. Moreover, the CaF_2_-Na_2_O-Al_2_O_3_-CaO-SiO_2_-MgO isoviscosity diagram at 1773 K was drawn through this model estimation and experimental detection, and the CaF_2_ mass fraction was close to 14% in the low viscosity zone.
3. The viscosity model and isoviscosity diagram were modified according to the XRF analysis of slag after the viscosity test. After contrast and analysis, it was found that Na_2_O and CaF_2_ decreased significantly, and the measured viscosity was larger than the theoretical viscosity due to mold flux volatilization.
4. The effect of volatilization on slag viscosity is significant. Therefore, it is necessary to conduct further research on the volatilization mechanism to control the physicochemical properties of slag and obtain a more accurate viscosity estimation model.

## Supporting information

S1 File (RAR)
