# Supplementary material for: Viscosity estimation model of fluorine-containing mold flux for continuous casting
Source: PLoS One. 2021 Mar 5;16(3):e0247828. doi: 10.1371/journal.pone.0247828 (PMC7935324; doi:10.1371/journal.pone.0247828)
Supplement: Attachment — Submitted filename: Response to Referees.docx [file pone.0247828.s002.docx]

Response to Referees Letter

Dear Editor,

Thank you for your letter that the manuscript entitled " **Viscosity Detection and Estimation Model of Fluorine-containing Mold Flux for Continuous Casting** " was received according to the editorial department's opinion.

Thanks to the two reviewers for their comments and suggestions. We have modified this article and answered all the questions one by one, as shown below.

Reviewer #1:

**1. Abstract：**

Please shorten the abstract (avoid redundant words).

Avoid abbreviations in the abstract/conclusions.

Avoid redundant sentences/words.

Avoid beginning a sentence (especially a paragraph) with conjoining words. e.g. For, As, When, etc.

**Response:** Thank you for Reviewer’s suggestion and the authors make modification and supplement, as shown in the text.

**2.** **Figures and** **Tables:**

Captions should have stand-alone meaning.

Quality should be improved

**Response:** Thank the Reviewer for the reminder and the captions and quality of figures have been modified.

**3. Writing style**

Do not repeat the heading in the text of the body.

**Response:** The expression in the text has been simplified.

Redundant words/sentences/ Unclear sentences.

Please leave a space between the number and scientific units.

The clear and concise writing style is required.

Please try to shorten the text by combining sentences, without losing the meaning.

Remove any redundant words or sentences.

**Response:** In view of the above format problems, modifications and corrections have been made in this paper.

**4. Originality:**

Please make it clear if there is any connection to this article: <https://pubs.acs.org/doi/10.1021/acs.energyfuels.0c00926>

**Response:** This article has no reference relationship with this article.

The paragraph just after Fig.4 is somewhat matching with this: <https://link.springer.com/book/10.1007%2F978-3-319-48769-4>

**Response:** The paragraph has been supplemented.

**5. Literature survey:** **Should be strengthened (very short) and no new references, e.g., 2020**

**Response:** New reference has been added in the article.

**6. Conclusions:**

It should be "Conclusions".

Please shorten the conclusions. Clear and concise and explicit jargon should be used.

**Response:** Thank the Reviewer for the reminder and the conclusions have been revised.

**7. Recommendation: The article needs major revision before considering for publication. The article is too short.**

**Response:** Thanks to the valuable comments, and we have made a deeper analysis of this viscosity model in the article.

Reviewer #2:

**1.** **In the Experimental part, the temperature range in which the viscosity was determined is not mentioned. It is assumed, according to Fig.1 that it is between 1100-1400ºC. But experimental data at 1773 K (1500ºC) are reported in Fig. 5 (from where were the experimental data in Fig. 5 taken?).**

**Response:** Thanks for the reminder and the experimental conditions have been supplemented in the paper.

**2. It should be clarified that the compositions of samples M1 to M11 (Table 2) the wt% Na2O corresponds to the sum of wt% Na2O and wt% K2O of the original work (Reference 20).**

**Response:** Thanks to the reminder and it has been supplemented and noted at the end of Table 2 in the text.

**3. The X-axis of Fig.1 should be 1/T, but it is labeled ln (1/T). (Should be corrected).**

**In the Y axis of Fig.1 it is necessary to mention the viscosity units (Pa.s?). Please, complete.**

**Response:** Thanks to the reviewer's reminder and we have made corrections.

**4. In the same Fig. 1 the parameters: lnA (= Intercept = -10.7828) and B (= Slope = 13602.93) correspond to sample C1 (according to Table 3), and not to sample C27 as it is mentioned in the text.**

**Response:** Thank you very much for your reminder, and it has been corrected in this paper.

**5. The expressions for the calculation of lnA and B should be revised. They do not coincide with those analyzed in the tables, according to the chemical composition.**

**Response:** Thanks to the valuable opinions. We have given a deeper analysis and discussion of this model in Fig. 2, which showed the influence of the components changes on viscosity graphically and intuitively.

**6. The section: "Application of this viscosity estimation model", should be discussed more extensively and in greater depth.**

**Response:** Thank you for your interest and a deeper analysis of this section has been supplemented under Fig. 5 in this paper.

**7. In line 108, it is mentioned: "When the CaF_2_ content was more than 17%, the viscosity decreased with the addition of Al_2_O_3_". However, this effect is not shown in Fig. 4. where, for CaF_2_ <17 wt% the viscosity always increases with increasing Al_2_O_3_ addition.**

**Response:** Dear reviewer, it's my honor that this research can arouse your interest, and the interpretation of Fig.4 is as follows: There are 6 variables involved in the slag system, therefore, take C27 as the basis, the MgO (wt%) and alkalinity keep constant, it can be obtained with influence on the slag viscosity by changing the CaF_2_, Na_2_O and Al_2_O_3_ components.

It can be concluded from Fig. 5(a) that the CaF_2_ content is 17% at the intersection point. On the left side of the intersection, i.e. CaF_2_(wt%)<17%, the increase in Al_2_O_3_ significantly increased the viscosity of the slag. When the CaF_2_ content was more than 17 %, the viscosity decreased with the addition of Al_2_O_3_. The same is true for other parts of Fig. 5.

**8. It would have to provide more details on the calculation methodology to obtain Fig.5 and Fig.6.**

**Response:** Thanks to the valuable suggestions and we have supplemented the ideas and methods of drawing the isoviscosity diagram in this paper.
