# Supplementary material for: Viscosity estimation model of fluorine-containing mold flux for continuous casting
Source: PLoS One. 2021 Mar 5;16(3):e0247828. doi: 10.1371/journal.pone.0247828 (PMC7935324; doi:10.1371/journal.pone.0247828)
Supplement: Attachment — Submitted filename: Response to Reviewers.docx [file pone.0247828.s003.docx]

Response to Reviewers Letter

Dear editor and reviewers,

Thank you for your letter that the manuscript entitled " **Viscosity Estimation Model of Fluorine-containing Mold Flux for Continuous Casting** " was received according to the academic opinion and guidance.

On the one hand, we accept the proposal that the word detection has been removed from the paper title and from the title of section “Viscosity detection and analysis”. On the other hand, we have modified the introduction to summarize and compare the previous research, and highlight the novelty and application of the work.

Thanks to the guidance and support from all editors and reviewers. We are willing to share more research to PLOS ONE in the near future.

Kind regards,

Jun-xue Zhao

Xi'an University of Architecture and Technology
